# Primary glomus tumour of the pituitary gland: diagnostic challenges of a rare and potentially aggressive neoplasm

**DOI:** 10.1007/s00428-020-02923-4

**Published:** 2020-09-12

**Authors:** Boon Leong Quah, Carmine Antonio Donofrio, Stefano La Rosa, Jean-Philippe Brouland, Giulia Cossu, Ibrahim Djoukhadar, Helen Mayers, Patrick Shenjere, Marta Pereira, Omar N. Pathmanaban, Muhammed O. Murtaza, Rao Gattamaneni, Federico Roncaroli, Konstantina Karabatsou

**Affiliations:** 1 Department of Neurosurgery, Manchester Centre for Clinical Neurosciences, Salford Royal NHS Foundation Trust, Manchester Academic Health Science Centre, Manchester, UK; 2 Division of Neuroscience and Experimental Psychology, Faculty of Biology, Medicine and Health, Manchester Centre for Clinical Neuroscience and Manchester Centre for Clinical Neuroscience, Salford Royal NHS Foundation Trust, Manchester Academic Health Science Centre, Manchester, UK; 3 Institute of Pathology, University Hospital and University of Lausanne, Lausanne, Switzerland; 4 Department of Neurosurgery, University Hospital of Lausanne, Lausanne, Switzerland; 5 Department of Radiology, Manchester Centre for Clinical Neurosciences, Salford Royal NHS Foundation Trust, Manchester Academic Health Science Centre, Manchester, UK; 6 Department of Cellular Pathology, Salford Royal Foundation Trust, Salford, Manchester UK; 7 Department of Cellular Pathology, Christie NHS Foundation Trust, Manchester, UK; 8 Manchester Centre for Genomic Medicine, St Mary’s Hospital, Division of Evolution and Genomic Science, University of Manchester, Manchester, UK; 9 Department of Endocrinology, Ysbyty Gwynedd, Bangor, Wales UK; 10 Department of Clinical Oncology, Christie NHS Foundation Trust, Manchester, UK

**Keywords:** Pituitary gland, Sella, Non-neuroendocrine tumour, Glomus tumour, Malignant

## Abstract

Primary non-neuroendocrine tumours of the pituitary gland and sella are rare lesions often challenging to diagnose. We describe two cases of clinically aggressive primary glomus tumour of the pituitary gland. The lesions occurred in a 63-year-old male and a 30-year-old female who presented with headache, blurred vision and hypopituitarism. Neuroimaging demonstrated large sellar and suprasellar tumours invading the surrounding structures. Histologically, the lesions were characterised by angiocentric sheets and nests of atypical cells that expressed vimentin, smooth muscle actin and CD34. Perivascular deposition of collagen IV was also a feature. Case 2 expressed synaptophysin. INI-1 (SMARCB1) expression was preserved. Both lesions were mitotically active and demonstrated a Ki-67 labelling index of 30%. Next-generation sequencing performed in case 1 showed no mutations in the reading frame of 37 commonly mutated oncogenes, including *BRAF* and *KRAS*. Four pituitary glomus tumours have previously been reported, none of which showed features of malignant glomus tumour. Similar to our two patients, three previous examples displayed aggressive behaviour.

## Introduction

Primary non-neuroendocrine tumours of the pituitary gland and sellar region are rare and are often challenging to diagnose preoperatively and at pathological examination. If craniopharyngiomas and meningiomas are excluded, the remaining 1% of sellar non-neuroendocrine neoplasms includes the group of TTF-1 expressing tumours of the posterior pituitary, neuronal and paraneuronal, germ cell, haemopoietic, melanocytic, sellar salivary gland-like tumours, the spectrum of mesenchymal neoplasms including chordoma, and bone and cartilage tumours, sellar atypical teratoid rhabdoid tumour (ATRT), perivascular epithelial cell neoplasm (PEComa) and glomus tumour (GT) [1–4].

Glomus tumour is a perivascular neoplasm that occurs more commonly in the distal extremities and more often involving the nail bed where glomus bodies are present. Glomus tumours have also been documented in visceral organs such as the lung, stomach, pancreas, liver, gastrointestinal and genitourinary tract [5, 6]. The pituitary gland is an uncommon site for GT [7–10] where it probably derives from the thick tunica media of hypophyseal portal vessels that are similar to glomera of other sites and function as sphincter to regulate blood flow [7, 11, 12]. Histologically, GTs resemble the normal glomus bodies; glomangioma and glomangiomyoma represent morphological variants. Malignant GT (mGT) and GT with uncertain malignant potential (GT-UMP) have rarely been documented, predominantly in deep-seated locations [6, 13].

We describe two patients with primary pituitary GT presenting as large, invasive sellar and suprasellar lesions, one with a fluctuating clinical behaviour due to tumour apoplexy and the second with a short history of visual loss and amenorrhoea.

## Case presentations

### Patient 1

**Fig. 1:**
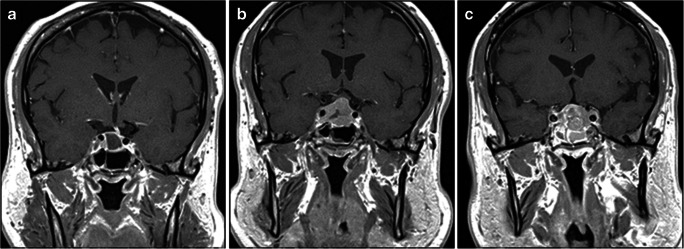
(Patient 1) Preoperative coronal post-contrast T1 MRI documents spontaneous resolution of the sellar lesion with a residual largely sellar cystic lesion causing deviation of the pituitary stalk to the left. There is minimal bulging into the suprasellar cistern but no compromise on the optic chiasm (A). Coronal post-contrast T1 imaging after 4 months shows interval deterioration in appearances with a recurrent solid enhancing sellar mass invading the right cavernous sinus. There is also worsening extension into the suprasellar cistern with displacement of the optic chiasm superiorly (B). Post-contrast coronal MRI after surgery and radiotherapy shows a recurrent solid enhancing sellar mass invading the cavernous sinuses bilaterally and with suprasellar extension (C)

A 63-year-old male sought clinical attention in 2008 for gynaecomastia without galactorrhoea. Hormonal tests revealed normal prolactin, growth hormone deficiency and hypogonadotropic hypogonadism, for which he was commenced on replacement therapy. Magnetic resonance imaging (MRI) at that time documented an expanded sella and a bulky pituitary gland but no tumour was observed. He presented again in August 2018 with worsening intermittent headaches and blurred vision. During that admission, he was diagnosed with adrenal insufficiency and hypothyroidism. Replacement therapy with hydrocortisone and levothyroxine was therefore administered. His headaches gradually deteriorated and developed blurred vision. Visual fields were full with no evidence of ophthalmoplegia. Investigations demonstrated worsening of anterior hypopituitarism and additional adrenal insufficiency. An MRI demonstrated a sellar lesion with suprasellar extension, left deviation of the stalk and slight optic chiasm displacement without compression. The patient was referred for pituitary surgery but the preoperative MRI showed spontaneous volumetric reduction with features consistent with apoplexy (Fig. 1a). Headaches and visual disturbances resolved completely. Surgery was therefore deferred. In June 2019, he returned with severe headaches and diplopia. On examination, he had right sixth cranial nerve palsy. A new MRI showed considerable enlargement of the lesion with suprasellar extension, mild compression of the optic chiasm and extension into the right cavernous sinus. There was also an increased solid enhancing component and a small area of central necrosis (Fig. 1b). The assessment of the hormonal axis at his local hospital revealed hypopituitarism. A whole-body CT scan was unremarkable.

The patient underwent endoscopic transsphenoidal surgery. Intraoperatively the tumour appeared firm and rubbery with a central area of relatively avascular, soft tissue consistent with necrosis. The sellar floor was eroded; the tumour was attached to the right side of the sphenoid septum. A residuum was left in the right cavernous sinus. Headache and diplopia resolved. He presented again 6 weeks later with diplopia and deteriorating visual acuity. An MRI showed a regrowth of the sellar and suprasellar components causing optic chiasm displacement. Further surgery was felt inappropriate and he was referred for adjuvant radiotherapy, which was completed in November 2019. A follow-up MRI documented shrinkage of the lesion following radiotherapy. He was re-admitted in May 2020 with complete third nerve palsy and sudden visual failure. An MRI demonstrated tumour progression with further invasion of the cavernous sinuses and involvement of the anterior cranial fossa (Fig. 1c). Further surgery and radiotherapy were considered unfeasible given the extent of the tumour and the patient has been referred for chemotherapy.

### Patient 2

**Fig. 2:**
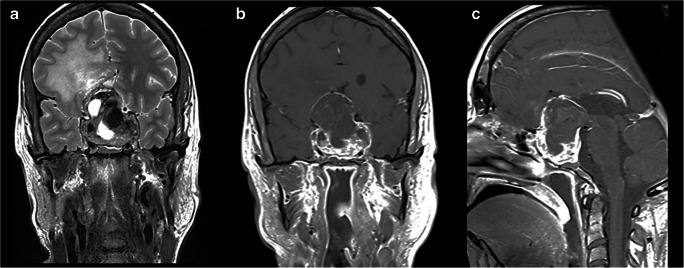
(Patient 2) MR imaging at presentation including (A) coronal T2, (B) post-contrast coronal T1 and (C) post-contrast sagittal T1 sequences documents a large heterogeneous mass lesion with heterogeneously enhancing solid and cystic components and intra-tumoural haemorrhage (A&B). The mass expands the sella and invades into the cavernous sinuses bilaterally. There is marked suprasellar extension and compression of the optic chiasm. The tumour bulges into the anterior aspect of the third ventricle (C) with secondary oedema and high T2 signal in the right frontal lobe (A)

A 30-year-old female presented with a 10-month history of progressive bilateral visual deterioration and amenorrhoea. She initially refused an endocrinological assessment and a brain MRI. She later sought clinical attention for a week history of severe headache and vomiting, suggesting acute increased intracranial pressure or apoplexy. Clinical examination showed complete bilateral blindness with optic disc atrophy. Oculomotor movements were preserved. A brain MRI showed a solid cyst, heterogeneously enhancing, intra- and suprasellar lesion with pituitary fossa enlargement, optic chiasm compression, third ventricle extension and bilateral cavernous sinus invasion. Oedema of the frontal lobes was also present (Figure 2a, b, c). Hormonal tests documented hypopituitarism and hyponatraemia. She was given hydrocortisone and levothyroxine and low sodium was corrected with hydric restriction.

The patient underwent an endoscopic endonasal transsphenoidal approach. Intraoperatively, the tumour was firm and difficult to remove. A subtotal resection was accomplished with decompression of the optic chiasm. In the postoperative course, she developed meningitis from *Candida Albicans*, with secondary hydrocephalus that required an external ventricular drainage and then a ventriculoatrial shunt. She remained hypopituitary on replacement therapy. She refused gamma knife treatment and decided for transcranial surgery in another centre. Surgery was complicated by a suprasellar haematoma, subarachnoid haemorrhage and stroke. She developed aphasia, right-sided hemiparesis and left-sided crural paresis and died 6 months after the second operation.

## Methods

The specimens were formalin fixed and embedded in paraffin. Five-micron thick sections were stained with haematoxylin-eosin (H&E). Immunohistochemistry was performed on a BenchMark ULTRA Slide Staining System (Roche Diagnostics, Indianapolis, IN, USA) using the primary antibodies directed against synaptophysin (Novocastra, monoclonal 27G12; dilution 1:500), FSH (Cell Marque Darmstadt Germany, monoclonal 83/122A8; dilution 1:2000), LH (Cell Marque, monoclonal 3LH5B6Y; dilution 1:100), TSH (Cell Marque, monoclonal 5404; dilution 1:50), PRL (Biogenex, monoclonal BGX031A; dilution 1:50), ACTH (Dako Agilent, monoclonal, clone 02A3; dilution 1:1000), GH (Cell Marque, monoclonal 54/92A2; dilution 1:50), steroidogenic factor 1 (Abcam Cambridge UK, monoclonal EPR19744; dilution 1:150), PIT1 (Novus Biologicals Abingdon Oxon UK, polyclonal; dilution 1:100), TPIT (Atlas Antibodies, Stockholm Sweden; monoclonal CL6251; dilution 1:300), pancytokeratin (Novocastra Newcastle UK; cocktail monoclonal AE1 and AE3; dilution 1:50), cytokeratin 7 (Dako Agilent Stockport UK, monoclonal OV-TL 12/3, dilution 1:50), cytokeratin 8 (Becton Dickinson San Jose CA, monoclonal CAM5.2; dilution 1:50), TTF-1 (Dako Agilent, 8G7G3/1; dilution 1:100), vimentin (Dako Agilent, monoclonal SRL-33; dilution 1:200), smooth muscle actin (Dako Agilent, monoclonal M0851; dilution 1:500), desmin (Dako Agilent, monoclonal M0760; dilution 1:100), myogenin (Dako Agilent, monoclonal M3559; dilution 1:500), CD21 (Dako Agilent, monoclonal M0784l; dilution 1:100), CD23 (Leica Biosystems, Milton Keynes UK, monoclonal NCL-L-CD23-1B12; dilution 1:50), CD31 (Dako Agilent, monoclonal M0823; dilution 1:30), CD34 (Dako Agilent, monoclonal M7165; dilution 1:50), ERG (Roche RTU; pre-diluted), Collagen IV (Roche RTU; pre-diluted), STAT6 (Sigma Aldrich, Gillingham UK, rabbit polyclonal, SAB4300331; dilution 1:100), GFAP (Dako Agilent, monoclonal 6F2; dilution 1:1000), S-100 protein (Dako Agilent; polyclonal; dilution 1:1000), p63 (Biocare, Pacheco CA, monoclonal 4A4; dilution 1:100), HMB45 (Dako Agilent, monoclonal M0634; dilution 1:50), PAX8 (Proteintech, polyclonal; dilution 1:250), alpha-fetoprotein (Dako Agilent, rabbit polyclonal, dilution 1:500), INI-1 (Cell Marque, monoclonal MRQ27; dilution 1:500), BRAFV600E (Ventana ,VE1 clone; pre-diluted), CD68 (Dako Agilent, clone KP1; dilution 1:200) and TFE3 (Roche RTU, polyclonal, pre-diluted).

The Ki-67 (Dako Agilent; monoclonal MIB-1, dilution 1:100) proliferative index was evaluated by counting the number of positive cells in at least 500 tumour cells in hotspot areas on camera-captured printed images.

### Next-generation sequencing

The most representative tumour area of case 1 was circled on a reference H&E-stained section. Five serial 10-micron thick sections were cut from formalin-fixed and paraffin-embedded tissue for DNA extraction. Genomic DNA was isolated from the selected tissue area using the cobas® DNA Sample Preparation Kit (Roche Diagnostics Ltd Burgess Hill UK). Sequence analysis was carried out following PCR enrichment using a QIAseq Targeted DNA custom panel (Qiagen, Germantown, MD, USA) and Illumina next-generation sequencing (Illumina, San Diego CA, USA). The panel included *AKT1*, *ALK*, *AR*, *ATRX*, *B2M*, *BRAF*, *CDKN2A*, *CTNNB1*, *DDR2*, *EGFR*, *ERBB2*, *FGFR3*, *GNA11*, *GNAQ*, *GNAS*, *H3F3A*, *H3F3B*, *HIST1H3B*, *HIST1H3C*, *HIST2H3C*, *HRAS*, *IDH1*, *IDH2*, *KIT*, *KRAS*, *MAP2K1*, *MET*, *NRAS*, *PDGFRA*, *PIK3CA*, *PTEN*, *RET*, *STK11*, *TERT*, *TP53* and *VHL*. The whole coding sequence was not targeted. Mutation and variant calling by custom bioinformatics analysis pipeline validated to detect SNVs and small insertion/deletion mutations (< 40 bp) to 5% mutant allele frequency. Alterations were categorised following the American College of Medical Genetics guidelines and Association for Molecular Pathology tiering.

### Pathological findings

**Fig. 3:**
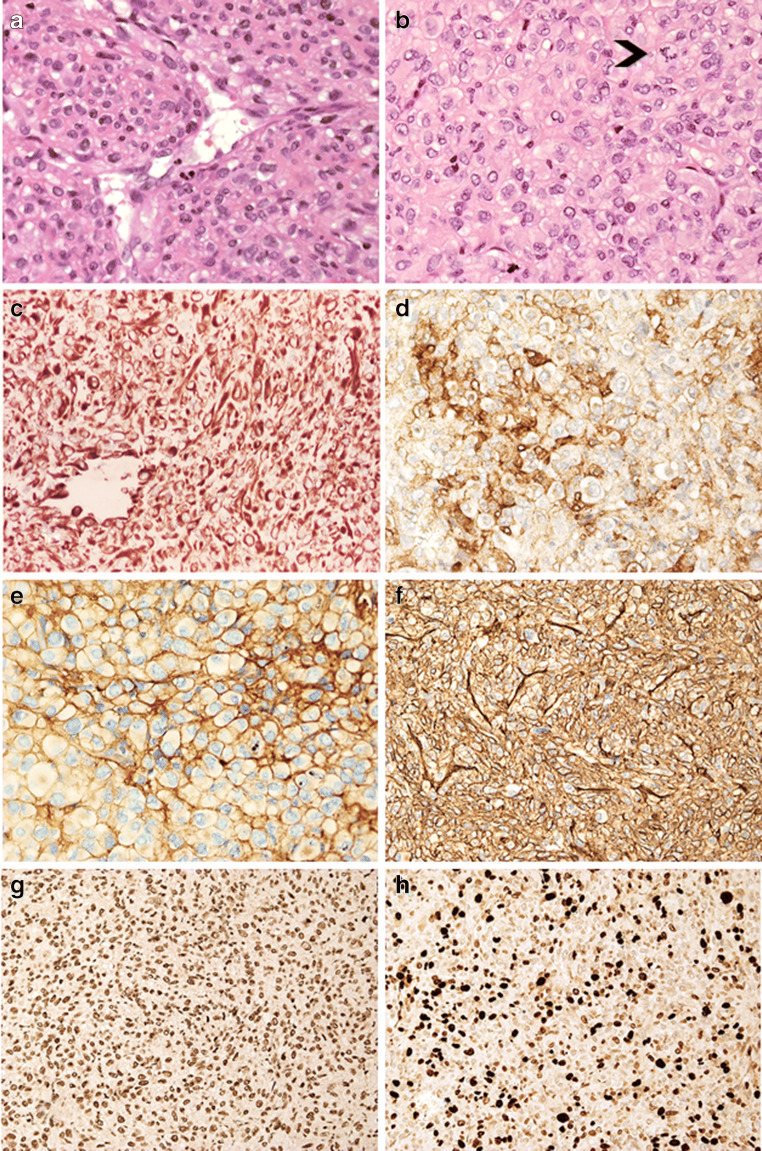
(Patient 1) The lesion is composed of densely cellular sheets of tumour cells that form thin-walled vessels (A, HE, × 20). Neoplastic cells have epithelioid features and display eosinophilic cytoplasm and hyperchromatic nucleus; atypical mitoses are also present (arrowhead) (B, HE, × 40); vimentin is diffusely expressed (C, immunoperoxidase × 20); smooth muscle actin is focally expressed in tumour cells (D, immunoperoxidase, ×40); there is pericellular deposition of collagen IV (E, immunoperoxidase, × 40); tumour cells expressed CD34 (F, immunoperoxidase × 20) and retain INI-1 nuclear expression (G, immunoperoxidase × 20). About 30% of tumour cells express Ki-67 (H, immunoperoxidase, × 20)

**Fig. 4:**
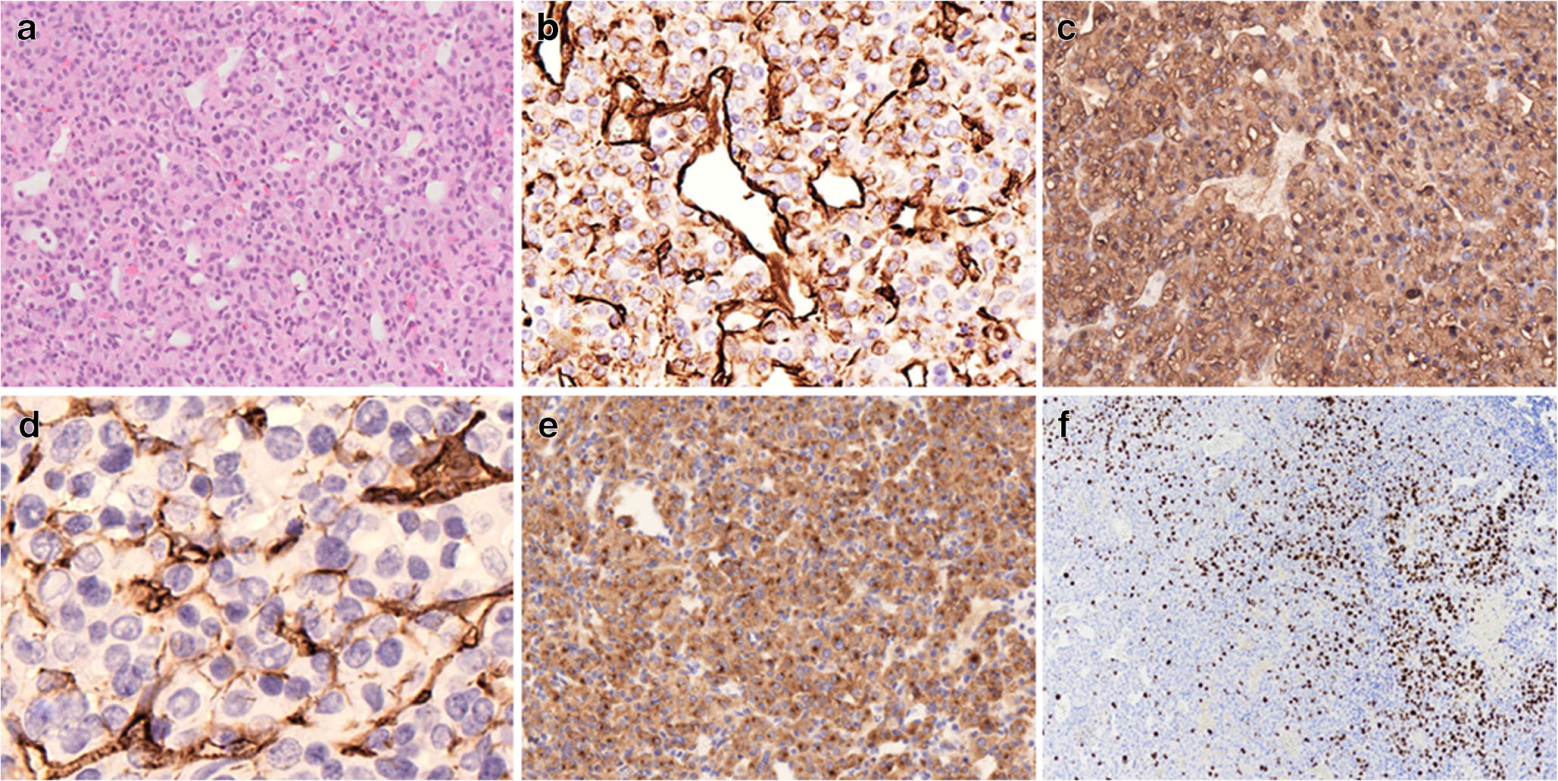
(Patient 2) The tumour is composed of sheets of atypical cells in a network of thin-walled vessels (A, × 20 – HE); neoplastic cells show diffuse expression of vimentin (B, immunoperoxidase × 20) and smooth muscle actin (C, immunoperoxidase × 20), focal CD34 (D, immunoperoxidase × 40) and diffuse synaptophysin (E, immunoperoxidase × 20); increase in Ki-67 is focal with areas showing up to 30% of positive tumour cells (F, immunoperoxidase × 10)

Both tumours showed moderate to high cellularity and consisted of nests, sheets and ill-defined lobules in a dense network of thin-walled vessels. Neoplastic cells were of small to medium size and showed neat borders, densely eosinophilic cytoplasm and irregular nucleus with fine chromatin and inconspicuous nucleolus. Several nuclei contained optically clear inclusions. Cytoplasmic vacuoles were present in some cells. Tumour cells often seemed to arise from or form themselves vessel walls (Figures 3a, 3b, 4a). No adeno- or neurohypophysis was present in the specimens. Mitotic count was up to 10 × 10 HPFs at × 400 magnification in case 1 and 2 × 10 HPFs in case 2. Lesion 1 also showed focal necrosis and haemorrhagic changes.

Neoplastic cells of both lesions expressed vimentin, SMA, which was focal in case 1 and diffuse in case 2 and CD34 that was focal in case 2; the immunoreaction for collagen IV showed pericellular pattern. Synaptophysin was only expressed in case 2. Scattered tumour cells in case 1 demonstrated membranous EMA immunoreactivity. SMARCB1/INI1 was normally expressed, CD68 highlighted macrophages and CD31 highlighted the dense vascular network. All other immunostains were negative. The Ki-67 labelling index was 30% in both tumours (Figures 3c, d, e, f, g and h and 4b, c, d, e and f).

The NGS panel did not identify any mutations in the reading frame of the 37 genes tested.

## Discussion

We have documented two primary GTs of the pituitary presenting as a large, invasive sellar and suprasellar masses. Both patients underwent repeated surgeries.

Given the site of the two lesions, the diagnosis of GT in our patients was challenging. Angiocentric sheets of tumour cells expressing vimentin, SMA and CD34 were important clues to reach the diagnosis of GT [5, 6]. The vascular network was not prominent enough to suggest glomangioma, no smooth muscle cells were identified to suggest glomangiomyoma and no bizarre cells were present for the symplastic variant of GT. Similar to our cases, GTs typically express vimentin and SMA [6], while CD34 has only been reported in some cases [9, 14]. Pericellular deposition of reticulin fibres and collagen type IV is also a common feature of GT and it highlights pericellular basal membranes. Neoplastic cells of case 2 expressed synaptophysin similar to the sellar GT documented by Tsang and colleagues and to visceral examples [10]. Our cases met the criteria of mGT [13] and showed malignant features from onset rather than during progression including high mitotic rate and high Ki-67 labelling index. Although the relevance of tumour size and extension is not established in pituitary GTs, the two lesions reported here were larger than 2 cm and locally invasive. It is accepted that designation of GT-UMP and mGT may not necessarily imply an aggressive clinical course; nevertheless mGTs recur frequently and have metastatic potential requiring a long-term follow-up and adjuvant treatment [13].

No mutations in the open reading frame of commonly mutated oncogenes, including *BRAF* and *KRAS*, were identified in our patient 1. Strong nuclear INI-1 expression in both lesions indicated that mutations in *SMARCB1* were unlikely to have occurred. The genetic features of GT, GT-UMP and mGT are not fully understood [15]. *BRAFV600E* and *KRAS* mutations were documented in superficially located GTs including GT-UMP and mGTs [16]. Dabek and colleagues found a stop gain mutation C > T in exon 6 and the frameshift truncation c.1148delC in exon 9 in *SMARCB1* [17]; unlike our cases, immunohistochemistry confirmed the loss of INI-1 expression. GTs have been described in neurofibromatosis type 1 [18, 19], but our patients had no stigmata and family history of the condition. No multiple GTs were identified to suggest an inherited condition such as mutations in the glomulin gene [20]. Rearrangements and novel fusion genes MIR143-NOTCH2 and MIR143-NOTCH1 were found in malignant GTs [21] but they were not tested in our cases.

Given the location of our cases, we considered a broad differential diagnosis. Synaptophysin expression seen in our case 2 and in one published example [10] could have misled to an erroneous diagnosis of aggressive immunonegative pituitary neuroendocrine tumour. The absence of lineage-restricted pituitary transcription factors along with vimentin, SMA, CD34 and collagen IV expression was not in keeping with a tumour that originated from adenohypophyseal cells. Paraganglioma, sellar melanocytic tumour, sellar ATRT, atypical meningioma, myoepithelial carcinoma, malignant solitary fibrous tumour, PEComa and epithelioid leiomyosarcoma were also considered in the differential diagnosis but light microscopic features and immunoprofile of the present lesions excluded these possibilities. Finally, we considered a mesenchymal neoplasm with a pericytic phenotype driven by fusions of *GLI1* with the partner genes *ACTB1*, *MALAT1* or *PTCH1* [22]. Histologically, tumours with *GLI1* aberrations show nests, cords and reticular structures of monomorphic round to epithelioid cells in a dense capillary network that can mimic GT. Unlike our GT, *GLI1*-fused tumours express S100 protein and are negative for SMA. A metastasis from a malignant GT was excluded clinically because no other lesion was found outside the sella.

Primary pituitary GT with features of glomangioma was first documented by Asa and colleagues in 1984 [7]. Since their report, other three cases have been published [8–10]. The essential clinical and pathological features of these four and our cases are summarised in Table 1. The previously documented pituitary GTs occurred in three adults aged 42, 47, 72 years [7–9] and an 8-year-old child [10]. Two were female [8, 10]. The tumours caused headache, visual impairment and intermittent diplopia. Two patients had panhypopituitarism, one at onset [10] and one after the second operation [10]. All tumours were sellar with suprasellar extension; cavernous sinus invasion was recorded in two cases [8, 9]. Similar to our patients, tumour removal was sub-total. Adjuvant radiotherapy was administered to three patients [7, 8, 10]. Three patients had multiple local tumour recurrences between 3 and 26 years from onset [7–10]. One patient died of tumour progression 12 years after the diagnosis [8]. Histologically, two lesions were described as glomangioma [7, 9] although these had predominant spindle cell morphology [9] leading to consider spindle cell oncocytoma, pituicytoma and meningioma in the differential diagnosis. A delicate fibre network surrounding individual tumour cells is a hallmark of pituitary GTs. SMA was described in all cases without any mention to focal expression as it was seen in our case 1. CD34 was tested in three cases and was negative in tumour cells. Synaptophysin was investigated in one lesion where it was found to be diffusely positive, in the absence of chromogranin and CD56 expression [10]. Electron microscopy was performed in three cases [7–9] and revealed medium-sized tumour cells featuring microvilli, pinocytotic vesicles, large mitochondria, myofibrils with focal condensation into dense bodies and basal lamina surrounding individual cells. Mitoses were described in two primary tumours without providing an accurate count [7, 10]. One case demonstrated increased cellular atypia and increased mitoses and Ki-67 at recurrence in keeping with a diagnosis of GT-UMP [10]. However, none of the previously documented cases presented with microscopic features of mGT in the primary lesion. *BRAFV600E* status was tested by immunohistochemistry in one instance [10]; otherwise, no genetic testing or immunostain for INI-1 was performed.

**Table 1:** Summary of the clinical and pathological features of primary pituitary glomus tumours

Case	Age/gender	Signs and symptoms at onset	Pituitary function	Tumour extension	Treatment	Outcome	Pathology
Asa et al., 1984 (Ref 6)	M/42	Decreased visual acuity; headache	N.A.	Sellar and suprasellar	Surgery (TC) RT	Recurrence 3 and 4 years	Glomangioma Ki-67: N.A.
Hänggi et al., 2005 Ref (7)	F/47	Intermittent diplopia	N.A.	Sellar and suprasellar; cavernous sinus	Surgery (TC); RT	Recurrence 8 and 10 years; DOD: 12 years	GT Ki-67: 10% at recurrence
Ebinu et al., 2011 (Ref 8)	M/72	Bitemporal hemianopia	N.A.	Sellar and suprasellar; cavernous sinus	Surgery (TS)	N.A.	Glomangioma Ki-67 > 10%
Tsang et al., 2020 (Ref 9)	F/8	Decreased visual acuity	Panhypopituitarism	Sellar and suprasellar	Surgery (TC); GK	Recurrence 4 and 26 years	GT-UMP Ki-67: 15% at recurrence
Patient 1	M/63	Headache; blurred vision (apoplexy)	Panhypopituitarism	Sellar and suprasellar; cavernous sinus; sphenoid sinus	Surgery; RT	10 months	mGT Ki-67: 30%
Patient 2	F/30	Headache; vomiting	Panhypopituitarism	Sellar and suprasellar; cavernous sinus	Surgery (TS/TC)	DOD 4 years	mGT Ki-67: 30%

*F* female, *M* male, *N.A.* not available, *TC* transcranial surgery, *TS* transsphenoidal surgery, *RT* radiotherapy, *GK* Gamma Knife radiosurgery, *GT* glomus tumour, *GT-UMP* glomus tumour with uncertain malignant potential, *mGT* malignant glomus tumour. *DOD* died of disease

## Conclusion

In conclusion, we documented two primary GTs of the pituitary gland displaying aggressive histological features at onset. Glomus tumour should be considered in the differential diagnosis of rare primary non-neuroendocrine tumours of the pituitary gland and sellar region as they present as invasive lesions that are difficult to remove surgically, have the tendency to recur locally, causing severe morbidity to patients, and require adjuvant treatment, predominantly radiotherapy as the evidence of efficacy of chemotherapy protocols remains very limited. Pituitary GTs can be diagnostically challenging, particularly when they show severe cellular atypia and high proliferation. As recommended by Tsang and colleagues [10], it is essential that the appropriate panel of immunostains that includes SMA and CD34 is requested to reach the diagnosis and inform on outcome.

## Data Availability

The full neuroimaging studies and histological slides of the two lesions are available for review.
